# Exposure to Nanoplastics and Co-Contaminants in Foods and Environment: Threats to Human Health

**DOI:** 10.3390/foods14193349

**Published:** 2025-09-26

**Authors:** Shuo Xiang, Mingyu Chen, Jun Liu, Jun Wang

**Affiliations:** 1 School of Civil Engineering and Architecture, Zhejiang University of Science and Technology, Hangzhou 310023, China; 2 Institute of Eco-Environmental Research, Guangxi Academy of Sciences, Nanning 530007, China; 3 College of Science and Technology, University of Macau, Macau 999078, China; 4 College of Marine Sciences, South China Agricultural University, Guangzhou 510642, China

**Keywords:** nanoplastics, combined pollution, toxicological effects, human health

## Abstract

Nanoplastics have emerged as significant global pollutants, drawing worldwide concern. Due to their small particle size, large specific surface area, and high surface activity, nanoplastics can combine with other environmental contaminants, including environmental nanoparticles, persistent organic pollutants, antibiotics, and endocrine-disrupting chemicals. This review summarizes recent progress on the environmental behavior of nanoplastics and their complex effects on food safety when co-exposed to various contaminants. These composite pollutants accumulate in foods and the environment, and are ultimately taken up by humans, posing potential toxic effects on human health. In the future, the interaction mechanisms between environmental NPs and various co-contaminants, as well as their transfer routes from food to humans, should be addressed.

## 1. Introduction

Since the 1950s, plastics have been widely used in packaging, construction, healthcare, and agriculture due to their excellent properties and low production costs. However, the massive production and improper disposal of plastic products have led to a continuous increase in their environmental residues, making plastic pollution an increasingly severe global issue [1].

Under the influence of natural forces and human activities, large plastic debris gradually degrades into microplastics (MPs, size of 1 µm−5 mm) and nanoplastics (NPs, with size generally < 1 µm) [2], which are contaminants of emerging concern in the environment. Beyond the fragmentation of environmental plastic debris, microplastics and nanoplastics (MNPs) in the environment also originate from inadequate disposal of specific products produced at a micro/nanoscale size [3]. MNPs were found in various foods originating from ocean, freshwater, and terrestrial environments [4]. MNPs can be generated from both conventional fossil-based plastics (e.g., polystyrene (PS), polyethylene (PE), polypropylene (PP), polyethylene terephthalate (PET), polyvinyl chloride (PVC), etc.) [5] and biodegradable plastics (e.g., polylactic acid (PLA), poly(ε-caprolactone) (PCL), etc.) [6].

Compared with MPs, the smaller NPs show enhanced mobility and bioavailability [7]. NPs have been detected in various environmental media, including water, sediment, soil, and air. The NPs transferred in the food web and those generated during food processing are accumulated in food products, which will eventually be taken up by humans, posing potential threats to food security and human health [8,9,10]. NPs can enter various organisms and cross critical physiological barriers, such as the placental barrier and blood–brain barrier, inducing adverse effects including inflammatory responses, immunosuppression, oxidative stress, and genotoxicity [11].

The physicochemical properties of NPs determine their environmental transport behaviors and fate. NPs exhibit strong adsorption capacities and act as vectors for environmental co-contaminants, such as heavy metals and persistent organic pollutants (POPs), consequently facilitating their diffusion and transportation in the environment [12,13]. Compared with single contaminants, NPs in combination with other environmental pollutants exhibit complex effects, including synergistic, antagonistic, and independent effects [14,15] on the risks of food security and human health. Furthermore, NPs and co-contaminants can enter the human body through food, drinking water, and air, potentially causing chronic toxicity effects across multiple organ systems. However, prospective studies on their complex effects on human health are still lacking.

This work reviews the latest progress on the food security and human health risks associated with exposure to NPs and environmental co-contaminants, emphasizing their complicated effects. Further research elucidating the interaction mechanisms of environmental NPs and various contaminants, as well as their transfer mechanisms from food to humans, will contribute to a deep understanding of their complicated effects on humans.

## 2. Environmental Behavior of Nanoplastics

### 2.1. Distribution and Transportation in Environments

**Figure 1:**
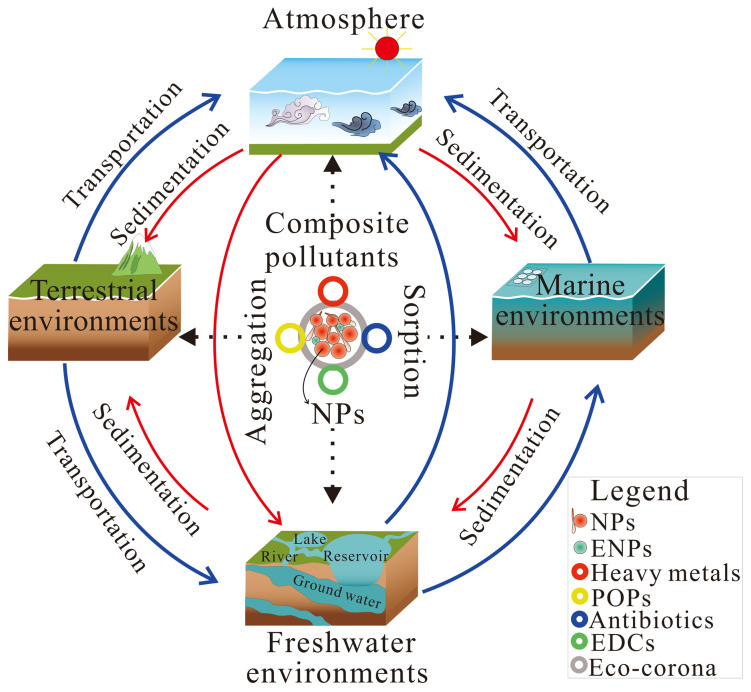
The possible distribution and transport of nanoplastics and co-contaminants in the environment. The term composite pollutants refers to nanoplastics (NPs) and co-contaminants, which include environmental nanoparticles (ENPs), persistent organic pollutants (POPs), and endocrine-disrupting chemicals (EDCs).

NPs are ubiquitously distributed in marine, terrestrial, and atmospheric sinks (Figure 1), such as seawater [16,17,18], sediment [19], river water [20,21], ground water [22], potable waters [23], ice [24], snow [25], air [26], soil [27] and even human brains [28].

The concentrations and polymer types of NPs vary in these sinks. For example, in the water column of the North Atlantic, about 1.5–32.0 mg m^−3^ of PET, PS, and PVC-NPs (filtered through a 1 µm PTFE syringe filter) were detected with thermal-desorption proton-transfer-reaction mass spectrometry (TD-PTR-MS) [18]. And the total mass concentrations of NPs (10 nm–1 μm) in surface water from the Fuhe River and groundwater, China were 0.283–0.793 μg L^−1^ and 0.021–0.203 μg L^−1^, respectively, by pyrolysis gas chromatography−mass spectrometry (Py-GC/MS) [22]. And NPs (<1 μm) were detected in bottled water at about 2.16 × 10^5^ particles per liter through a hyperspectral stimulated Raman scattering (SRS) imaging platform [29]. Also, for the Antarctic sea ice samples, the average concentrations of NPs (<200 nm) at the top and bottom were 67.0 and 37.7 ng/mL, respectively, based on TD-PTR-MS [24]. Further, NPs (10 nm–1 μm) in municipal water (0.55 and 0.61 µg L^−1^), bottled water (0.21 and 0.46 µg L^−1^), and reservoir water (1.5 µg L^−1^) from Australia were tested, and polymer types of PE, PET, PP, PS, polymethylmethacrylate (PMMA), and Nylon 6 were found by Py-GC/MS [23]. In addition, the concentration of NPs in surface snow after 0.2 μm filtration was 4.6–23.6 ng/mL, while the concentration of NPs in a snow pit at a depth of 2.8 m after 0.2 μm filtration was 18.5 ng/mL in snow samples, and only PET was found, while PET, polypropylene carbonate (PPC), and PVC were found before filtration by TD-PTR-MS [25]. While in a high-altitude alpine area, the concentration of NPs (<200 nm) in melted surface snow was 46.5 ng/mL, with a mean deposition rate of 42 kg km^−2^ year^−1^ detected by TD-PTR-MS [30]. And the determined concentrations of PS-NPs (<1 μm) were 6.5–8.5, 1.4–1.8, and 0.7–1.0 μg L^−1^ for water samples from a river, a mariculture farm, and a beach, respectively, by the optical manipulation–surface-enhanced Raman scattering approach [19]. The highest amount of NPs (<200 nm) was 180–1588 μg L^−1^, detected in freshwater at a Swedish sampling site analyzed by TD-PTR-MS [31]. Further, the highest concentrations of PS-NPs, 0.17 µg g^−1^ (<1 μm) in soil from Guangzhou and 0.73 µg g^−1^ in Pearl River sediment in Guangzhou, were detected by Py-GC/MS [32].

NPs can be transported far away from their source. The unique characteristics of NPs, such as their small size and buoyancy, enable them to remain suspended in water columns or be transported over long distances by wind and ocean currents [33]. In marine and freshwater systems, NPs can remain in suspension, aggregate with natural colloids, or settle into sediments depending on their density and environmental conditions [34]. In addition, NPs in soils may interact with clay minerals, organic matter, and soil microbiota, which affect their transportation in the terrestrial environment [35]. They can be transported via leaching into groundwater systems or taken up by plant roots, potentially entering terrestrial food webs [36,37]. Moreover, NPs can become airborne through wind erosion of contaminated soils or resuspension from water surfaces, and they can be transported globally, contributing to long-range environmental pollution [38]. The transportation of NPs is determined by their habits, physicochemical properties, and environmental behaviors, such as aggregation and sedimentation.

### 2.2. Aggregation and Sedimentation

Aggregation refers to the process by which NPs and environmental particles accumulate together and form larger clusters or aggregates. Their high surface-to-volume ratio makes NPs easily form homogeneous or heterogeneous aggregates [39], while in the natural environment, NPs usually undergo heteroaggregation because of their relatively low concentrations [40].

NPs can aggregate with various minerals (e.g., the clay colloids [41], coastal sand [17], and kaolinite [42]) and environmental nanoparticles (ENPs), such as nano-Fe_2_O_3_ [43], nano-TiO_2_ [44], and nano-GO [45]. For example, PS-NPs and nano-Fe_2_O_3_ aggregate at appropriate pH and relative abundances (0.1–0.4) [43]. Furthermore, NPs can heteroaggregate more minerals or organo-mineral-complex-coated sand than bare quartz sand [46].

The aggregation behavior of NPs in the environment is influenced by ionic strength, surface properties of NPs, natural organic matter (NOM), and environmental pH. For example, CaCl_2_ destabilizes both PE-NPs and PS-NPs more aggressively than NaCl and MgCl_2_, and PS-NPs are more stable in the aquatic environment than PE-NPs [47]. In addition, weathering of NPs alters their physicochemical properties and, consequently, their aggregation behavior [39]. For example, ultraviolet (UV) aged PS-NPs exhibited higher stability in a monovalent cation-dominated solution and lower stability in a divalent salt solution compared with sulfide-aged PS-NPs [48]. NPs are most stable in groundwater, then natural river water, and lastly seawater [40,41]. Moreover, at pH 5.0, antibiotics of both ciprofloxacin (CIP) and sulfamethoxazole (SMX) significantly promote nanoplastics aggregation, with the former being stronger through charge shielding and sulfamethoxazole through molecular bridging [49].

Furthermore, NOM is vital for the environmental behavior of NPs. NOM interacts with NPs rapidly when they enter aquatic environments. Generally, NOM could reduce the aggregation and sedimentation of NPs, exhibiting an excellent stabilization effect. While NOM, like humic acid, can promote the aggregation of PS-NPs when suspended sediments exist with or without NaCl, NaCl can also improve aggregation [50]. NOM and NPs interact through various mechanisms, including intermolecular forces [43] and the Ca^2+^ bridging effect [51]. And the interaction is affected by concentrations, sources [52], and properties of NOM [53]. Exposed to the natural environment, the surfaces of NPs are covered by various environmental coronas, including the ‘hard corona’ and ‘soft corona’ [54]. The ‘eco-corona’ modifies the particle sizes and surface properties of NPs [55,56], influencing their uptake by organisms and sedimentation. For example, the environmental corona on PS-NPs can alter their intracellular locations, activate new internalization pathways in both keratinocytes and fibroblasts, and modify cellular responses of keratinocytes [54].

Sedimentation occurs when aggregated NPs become sufficiently dense to settle onto the bottom of a water column [40]. The properties of NPs, such as polymer types and shapes, also affect their sedimentation in the environment. For example, with NOM, polymorphic NPs showed greater retention than spherical NPs in porous media [57].

### 2.3. Sorption and Carrier Effect for Co-Contaminants

NPs can sorb and carry various environmental co-contaminants (Figure 1), such as heavy metal ions, POPs (e.g., polycyclic aromatic hydrocarbons (PAHs) and polychlorinated biphenyls (PCBs), pesticides, etc.), antibiotics, and endocrine-disrupting chemicals (EDCs) [58]. Co-contaminants can attach to the surface of NPs by adsorption [59,60] or penetrate the solid matrix of NPs by absorption because of partitioning [61]. For example, lead (Pb) can be adsorbed onto NPs as complexes of monoligands and biligands [62]. And electrostatic and dispersion interactions dominate the adsorption of NP–neonicotinoid-insecticide complexes through physisorption onto the surface of all plastic (PET, PS, PE) matrices [63].

The adsorption capacities of NPs are affected by the physicochemical properties of NPs, such as the polymer type, size, and surface properties. For example, weathered PET, PS, and PP-NPs (<200 nm) showed strong adsorption of heavy metal ions, including Mn^2+^, Co^2+^, Zn^2+,^ and Cd^2+^, with PP-NPs showing the highest adsorption capacity [64]. The adsorption capacities of functionalized PS-NPs (i.e., PS-COOH-NPs, PS-NH_2_-NPs) were higher than those of pristine PS-NPs [65]. Compared with pristine PS-NPs, aged PS-NPs showed a higher adsorption capacity for heavy metals, such as Pb and cadmium (Cd) [39].

Also, as particle size decreases, the adsorption capacity of NPs increases, while the diffusion of co-contaminants inside NPs decreases [66]. For example, the adsorption capacity of NPs for butyltin increases with decreasing particle size and salinity [12]. And the adsorption capacity of 20 nm carboxylated PS-NPs for perfluorooctanoic acid (PFOA) is twice that of 500 nm PS-NPs in wastewater [67]. Also, the adsorption equilibrium constant (k_d_) for triclosan (TCS) increased from 1.36 L⋅g^−1^ of 900 nm PS-NPs to 4.39 L⋅g^−1^ for 50 nm PS-NPs [65].

The adsorption capacities of NPs are affected by the types of co-contaminants. For example, their adsorption of hydrophilic and negatively charged contaminants is lower than that of hydrophobic and cationic ones, and the adsorption capacity of PS-NPs for different co-contaminants follows glyphosate < PFOA < methyl parathion < phenanthrene < fluoxetine [67].

Environmental factors, such as salinity, pH [65], and NOM [68], can also affect the interactions of NPs and co-contaminants. For example, humic acid impedes the sorption of benzo[a]pyrene and copper (Cu) on anionic NPs, while facilitating that process on neutral and cationic NPs [69]. In addition, humic acid improved the sorption capacity for a certain antibiotic (ciprofloxacin, CIP) and endocrine disruptor (bisphenol-A, BPA) of 40 nm PS-NPs and 1 μm PVC-NPs [70]. A strong adsorption for sulfadiazine (SDZ) occurred when the relative abundance ratio of PS-NPs to nano-Fe_2_O_3_ at 0.2 was achieved through charge neutralization [43]. In addition, after aggregation with kaolinite, the PS-NPs showed an increase in the sorption capacity for Pb^2+^ [42].

### 2.4. Degradation and Persistence in the Environment

NPs are highly persistent in the environment due to their chemical stability and resistance to degradation. Degradation processes include photodegradation, oxidative degradation, and biodegradation, but these processes are generally slow in natural environments [55]. For photodegradation, UV radiation can break down NPs and fragment polymers into smaller particles. However, this process is limited in deep water and sediment environments where light penetration is low [71]. For biodegradation, the degradation efficiency of various microorganisms in natural environments, such as fungi and bacteria on NPs, is limited [55].

In addition, the degradation of biodegradable plastic is also limited. For example, although the PCL film is embedded with an enzyme, the released MPs and NPs after hydrolysis could not be completely degraded after up to 130 days [72]. Significantly, while the amorphous phase of the semicrystalline NPs continues to degrade, crystal fragments do not, and hence they temporally persist in the environment [73]. In addition, NPs are more stable and have longer residence times in freshwater compared with brackish water or seawater [40]. Furthermore, the presence of additives and stabilizers further complicates biodegradation [55].

## 3. Food Security and Human Health Risks of Nanoplastics and Co-Contaminants

### 3.1. Food Security Risks of Nanoplastics and Co-Contaminants

#### 3.1.1. Nanoplastics in Foods

**Figure 2:**
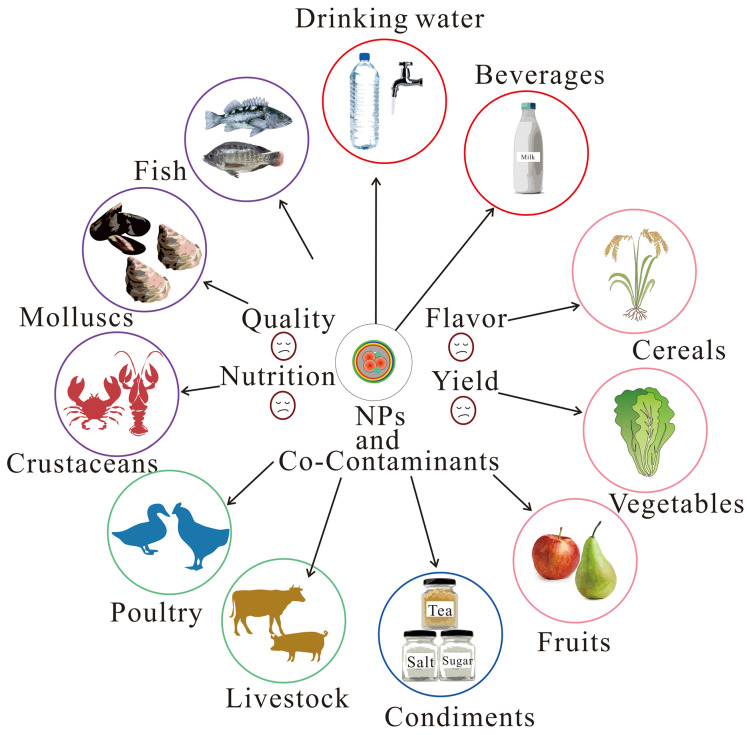
Risks of nanoplastics and co-contaminants on food security.

Food security is at risk due to NPs in the environment, food web, and food processing and food packing, which will eventually threaten the health of humans. Due to the small size of NPs and the detection limits of conventional spectroscopic techniques, detecting NPs is quite challenging [74], although simulated NPs in various foods, beverages, and condiments can be detected using Py-GC-MS or surface-enhanced Raman spectroscopy (SERS) methods [75]. However, the direct dictation of NPs in field samples of foods has not been reported yet, whereas the generation of NPs during food preparation and food packaging through various food contact articles, such as bottles, bags, and pans, has been verified [76,77,78]. These NPs can potentially enter into foods (Figure 2).

In the aquatic environment, crustaceans, mollusks, and fish are common food sources for humans. They are generally exposed to NPs by ingestion, which results in toxicological effects on their behavior, growth, and reproduction [79]. NPs can exacerbate oxidative stress, DNA damage [5], and neurotoxicity [80] in aquatic species, while also negatively impacting their behavior, antioxidant defense system, reproduction, and survival [81], and are associated with changes in crucial metabolic pathways [82]. The biological effects of NPs depend on their concentrations and physicochemical properties, such as size and surface charges [83].

Once exposed to the NPs, they could affect the nutritional quality of plants over time and gradually accumulate in fruits as plants grow, thereby reducing taste and flavor [84], and pose a risk to the security of foods [85].

NPs mainly accumulate in the root and leaves of crops [86,87]. NPs can enter plants by roots, apoplastic transport, foliar uptake, and transpirational pull [88]. For example, PS-NPs (50 nm) could enter the xylem vessel of the root and eventually be transported to the leaf, affecting the growth of pakchoi by disturbing the homeostasis of endogenous hormones [89]. In addition, PS-NPs (100–500 nm) can also transfer from root to shoot, accumulating in intercellular spaces and changing in phenolic compounds [90]. While PS-NPs (200 nm) primarily accumulated in the peels, cortex, and xylem of radish roots [91], they may be directly taken up by humans. Exposure to PS-NPs could trigger plant defense systems by upregulating gene expression and metabolites and downregulating genes associated with plant hormone signal transduction, which finally changes the nutrition and flavor of crops [91]. Moreover, NPs can be transferred to subsequent generations of pea plants (*Pisum sativum*) [92], indicating they can be continuously circulated in terrestrial ecosystems and have a lasting effect on food yield and quality. The toxicity of NPs can be affected by organism species, toxicity metrics, physicochemical properties of NPs, concentrations, exposure time, and medium.

NPs can affect the health of poultry and livestock. For example, when exposed to PS-NPs (100 nm) for 120 days, chickens showed histopathological liver injury with metabolic disturbances [93]. In addition, PS-NPs (100 nm) inhibited muscle fiber formation on porcine myoblasts derived from skeletal muscle satellite cells in vitro [94].

#### 3.1.2. Exposure to Nanoplastics and Co-Contaminants in Foods

Beyond NPs, foods are also exposed to various co-contaminants, such as phthalates and bisphenol A [95]. Exposure to NPs and co-contaminants has complex toxicity effects on aquatic food products, such as invertebrates, fish, and crops (Figure 2). Co-exposure to NPs and a single type of heavy metal, such as Cd, As, Pb, Cu, or Hg, has been frequently studied. For example, simultaneous exposure to PS-NPs (100 nm) and Cd synergistically led to more severe histopathological damage to liver tissue and increased liver transaminases activities of Prussian carp in 21 days, resulting in decreased superoxide dismutase (SOD) activity, with increased malondialdehyde (MDA) and total antioxidant capacity (T-AOC), and upregulated transcriptional levels of immune-associated genes [96]. PS-NPs could also enhance the bioaccumulation and toxicity of a heavy metal cocktail (HMC) in shrimp [97].

Exposure to NPs with different co-existing contaminants could cause different effects. For example, PS-NPs (100 nm or 80 nm) mitigate the intestinal toxicity of Cd [98] but aggravate the hepatopancreatic toxicity of phoxim (PHO) [99] and hepatotoxicity of perfluorooctanoic acid (PFOA) [100] in Chinese mitten crabs, which may be related to different interactions between NPs and co-contaminants.

Co-exposure to different kinds of contaminants for both freshwater fishes [101,102,103] and marine fishes [104,105] caused intestinal damage and hepatotoxicity, while fish intestines and liver usually being abandoned during food processing. Combined exposure to NPs and mercury (Hg) increased toxic effects on rare minnows, *Gobiocypris rarus*, compared with individual exposures, although NPs had limited effects on methylmercury (MeHg) content and its proportional distribution in muscle tissue of rare minnows [106], which may be linked to microbial dysbiosis with systemic metabolic dysfunction as well as oxidative stress and inflammatory responses triggered by NPs and/or Hg exposure.

Exposure to NPs and co-contaminants (e.g., heavy metals and POPs) also has a complex effect on crops [107], for example, increased bioaccumulation of PS (20 and 1000 nm) and As in edible tissues [108], as well as PS-NP (500 nm) and Cd [109] in shoots of lettuce, show increased effects in combination with smaller NPs [108] and saline conditions [110], which is related to an energy-intensive oxidative stress response [109]. In addition, although PS-NPs (100 nm) primarily accumulate in the roots of lettuce, they amplify the toxic effect of tebuconazole on auxin biosynthesis, inactivation, and signaling [111].

### 3.2. Human Health Risks of Nanoplastics and Co-Contaminants

**Figure 3:**
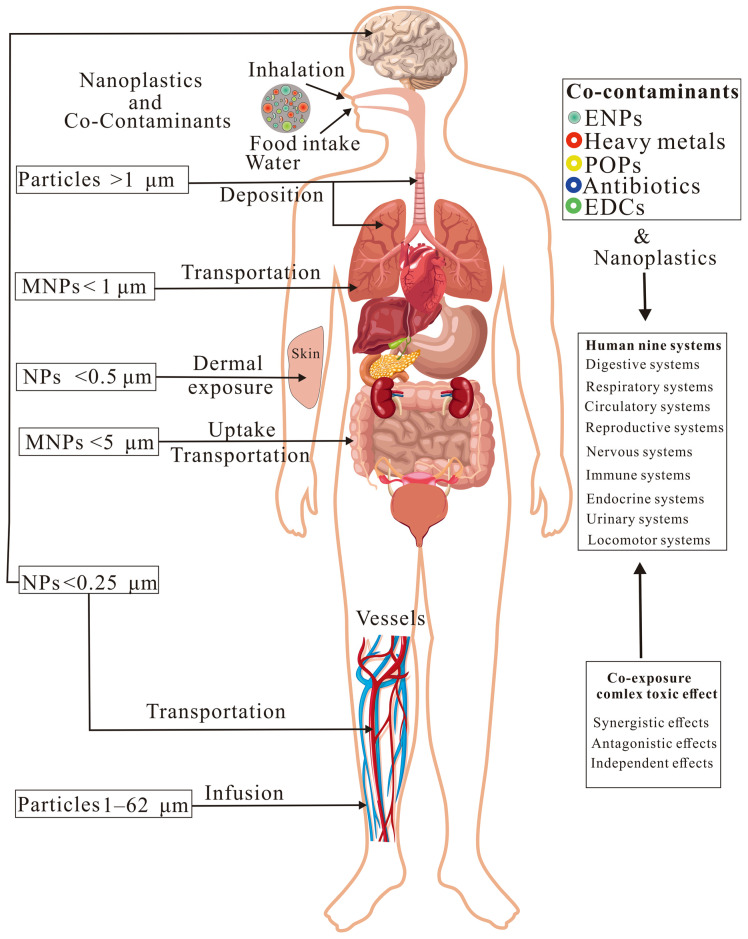
Potential health risks of nanoplastics and co-contaminants on humans. The terms composite pollutants refer to nanoplastics (NPs) and co-contaminants, environmental nanoparticles (ENPs), persistent organic pollutants (POPs), and endocrine-disrupting chemicals (EDCs).

Humans can be exposed to environmental NPs through ingestion, inhalation, and dermal exposure [112]. NPs in contaminated food and water can be ingested by humans, while airborne NPs can be inhaled or dermally absorbed by humans (Figure 3). NPs potentially pose a toxic effect on the human body through mechanisms such as oxidative stress, mitochondrial dysfunction, immune responses, and the gut–brain axis [113]. The potential toxic effects of NPs and co-contaminants have been shown on multiple physiological systems of humans.

#### 3.2.1. Ingestion and Infusions

Humans can ingest NPs through contaminated food and drinking water [80]. MNPs smaller than 5 μm can be directly taken up by intestinal epithelial Caco-2 cells [114]. In addition, MPs (1–62 μm, mainly PP, with NPs possibly existing) can be directly injected into the human bloodstream through infusions [115], which may deposit in various organs of humans. Once ingested, NPs can enter the gastrointestinal tract, where they may cross biological barriers, such as the intestinal lining, and possibly accumulate in the livers, kidneys, and even the brains of humans [28]. Similarly, PP and PET-NPs showed modest genotoxicity in DNA strand breaks, although they did not show significant cytotoxicity in human liver cancer cells HepG2 and intestinal epithelial cells Caco-2 [116]. However, NPs may change lipid digestion in the gastrointestinal system of humans, threatening the assimilation of nutrients [68].

A limited co-exposure study showed that PS-NPs (100 nm and 700 nm) and the heavy metals Cd, Pb, As, and chromium (Cr) synergistically increased the expression of inflammatory cytokines in the human intestinal epithelial cell line Caco-2 [117], indicating the ‘Trojan horse’ effects of NPs [118]. In addition, the combined toxicity of PS-NPs and perfluorooctanesulfonic acid (PFOS) on Caco-2 cells seems to be independent (no effects), with combined exposure disrupting RNA, endoplasmic reticulum, and mitochondria, with NPs as the primary driver of toxicity [119], which may indicate cross-talk between cellular structure and metabolism in response to the co-exposure.

#### 3.2.2. Inhalation and Dermal Exposure

Airborne NPs can be directly inhaled by humans and deposit in lung tissue, significantly influencing human respiratory health [120]. And 1 μm PS-NPs can be taken up by human lung alveolar A549 cells [121]. In addition, NPs can penetrate the epidermis and enter the human body through dermal exposure [122]. For example, after 16 h of incubation, fluorescently labeled PS-NPs (100 nm and 500 nm) were taken up by human skin keratinocytes and fibroblast cells [122]. PET-NPs internalized in the endosomes and nuclei of human primary nasal epithelial cells induced intracellular ROS induction and mitochondrial membrane potential loss [123]. In addition, both PVC-NPs and PS-NPs (250 nm) showed a strong affinity for phosphatidylcholines and lysophosphocholines from human plasma, with more PVC-NPs crossing the human blood–brain barrier than PS-NPs [124]. Also, amino-modified PS-NPs showed significant cytotoxicity on normal human dermal fibroblast cells, with the cytotoxicity decreasing as PS-NH_2_-NPs > PS-COOH-NPs > PS-NPs [125].

Co-exposure to NPs and other contaminants can have synergistic and antagonistic effects on human lung cells. For example, PS-NH_2_-NPs (68.6 nm) and spherical Nano-ZnO showed a synergistic effect on toxicity in human lung BEAS-2B epithelial cells, while PS-NH_2_-NPs and triangular pyramid of Nano-ZnO exhibited an antagonistic effect [126], owing to change in cell membrane integrity and oxidative stress. In addition, co-exposure to PET-NPs and cigarette smoke condensate (CSC) for 4 weeks synergistically exacerbated oxidative stress, genotoxicity, and tumorigenic transformation of human lung BEAS-2B cells compared with individual exposures [127], which may be attributed to enhanced carcinogenic traits through oxidative stress, genomic instability, and disruption of tumor-suppressive pathways.

#### 3.2.3. Potential Health Risks

Studies have shown that NPs can alter hormone levels by interacting with estrogen and thyroid hormone receptors. For example, NPs can affect the homeostasis of breast cells [128]. In addition, PS-Pd NPs can translocate into and interact with human primary immune cell subpopulations from human blood after exposure [129]. Furthermore, PS-NPs can influence the dysfunction of cellular components and the development of atrioventricular heart valves in human-induced pluripotent stem cells hiPSCs [130]. Furthermore, carboxylated PS-NPs (40 nm and 200 nm) affected early-pregnancy trophoblast phenotype and function of human trophoblast cells [131]. NPs can interfere with hormonal systems in humans, leading to endocrine disruption, which poses potential risks, especially for children and pregnant women, which can be potentially transferred to the next generation [132].

The ‘Trojan horse’ effect of NPs can increase the bioaccessibility of environmental contaminants to humans [133], enhancing their toxicity or even carcinogenic risk to humans [134]. For example, PS-NPs and the heavy metals Cd, Pb, As, and Cr synergistically increased the inflammation in human monocytic leukemia cell (THP-1)-differentiated macrophages [117]. PS-NPs (500 nm) significantly reduced the availability and uptake of Cd, reducing its cytotoxicity on human hepatocytes (HepG2) hepatoma cells in a dose-dependent manner [135], suggesting that cadmium might play an active role in the interaction between NPs and lipids, promoting tighter and more stable binding. In addition, co-exposure to NPs and the pharmaceutical and personal care product triclosan synergistically increased the production of reactive oxygen species (ROS) in human KGN ovarian granulosa cells [136], activating the antioxidant stress pathway. In addition, PS-NPs enhanced the binding of triclosan to human serum albumin, promoting its accessibility to the binding sites [137], which may be attributed to the alteration of human serum albumin conformation and microenvironment of the amino acid residues induced by NPs.

## 4. Research Trends

Although research on the ecological influence of NPs and co-contamination has made some progress, it remains in its infancy and faces numerous challenges. The direct detection of NPs in environmental samples of foods is still lacking, which indicates an urgent need for sound separation and analytical methods for NPs. Co-exposure studies are mostly conducted under laboratory conditions using a single type of NP and co-contaminant, which is different from environmental conditions, in which NPs are complicated, heterogeneous, and dispersed with long-term interaction.

Also, the study on how co-exposure to composite pollutants affects the taste, nutrition, and quality of various foods is still insufficient. In addition, the mechanisms of interaction between environmental NPs and novel pollutants and their molecular pathways on the aquatic and terrestrial organisms of food sources are underexplored.

Furthermore, studies evaluating human exposure to environmental NPs and co-contaminants are very limited. And studies are limited to co-exposure of human cells, making it difficult to achieve a deep understanding of the risks of NPs and co-contaminants to human health. Moreover, there are large gaps in evaluating exposure to NPs and co-contaminants through foods without looking into their transfer routes from foods to humans.

## 5. Conclusions

Environmental nanoplastics can sorb and aggregate with various environmental pollutants, forming composite pollution systems with enhanced mobility and bioavailability. Composite pollutants widely distributed in the environment can pose a risk to a variety of foods and will eventually be transported to humans. The carrier effects of NPs for co-contaminants are complicated and pose a risk of toxicity on food security and human health. Yet research on these complex effects is still in a very early stage and needs to be further explored.

## Data Availability

No new data were created or analyzed in this study.

## Abbreviations

The following abbreviations are used in this manuscript:

MPs	microplastics
NPs	nanoplastics
MNPs	microplastics and nanoplastics
PS	polystyrene
PE	polyethylene
PP	polypropylene
PET	polyethylene terephthalate
PVC	polyvinyl chloride
PLA	polylactic acid
PCL	poly(ε-caprolactone)
PMMA	polymethylmethacrylate
PPC	polypropylene carbonate
POPs	persistent organic pollutants
ENPs	environmental nanoparticles
EDCs	endocrine-disrupting chemicals
TD-PTR-MS	thermal-desorption proton-transfer-reaction mass spectrometry
Py-GC/MS	pyrolysis gas chromatography−mass spectrometry
SRS	stimulated Raman scattering
SERS	surface-enhanced Raman spectroscopy
NOM	natural organic matter
UV	ultraviolet
PAHs	polycyclic aromatic hydrocarbons
PCBs	polychlorinated biphenyls
PFOA	perfluorooctanoic acid
TCS	triclosan
CIP	ciprofloxacin
BPA	bisphenol-A
SDZ	sulfadiazine
IBU	ibuprofen
As	arsenic
Cd	cadmium
Cr	chromium
Cu	copper
Hg	mercury
Pb	lead
Zn	zinc
MeHg	methylmercury
DBDPE	decabromodiphenyl ethane
PCB-18	2,2’,5-Trichlorobiphenyl
DEHP	di(2-ethylhexyl) phthalate
PBDE-47	2,2′,4,4′-tetrabromodiphenyl ether
TDCIPP	tris (1,3-dichloro-2-propyl) phosphate
DES	diethylstilbestrol
APAP	acetaminophen
TPT	triphenyltin
DBP	dibutyl phthalate
Phe	phenanthrene
PYR	pyraclostrobin
F-53B	6:2 chlorinated polyfluorinated ether sulfonate
PFOS	perfluorooctanesulfonic acid
CSC	cigarette smoke condensate
CIP	ciprofloxacin
SMX	sulfamethoxazole
PHO	phoxim
HMC	heavy metal cocktail
SOD	superoxide dismutase
MDA	malondialdehyde
T-AOC	total antioxidant capacity
ROS	reactive oxygen species
